# Supplementary material for: Different Seasonal Collections of Ficus carica L. Leaves Diversely Modulate Lipid Metabolism and Adipogenesis in 3T3-L1 Adipocytes
Source: Nutrients. 2022 Jul 10;14(14):2833. doi: 10.3390/nu14142833 (PMC9323846; doi:10.3390/nu14142833)
Supplement: Supplementary file 1 [file nutrients-14-02833-s001.zip › nutrients-1769414-supplementary.pdf]

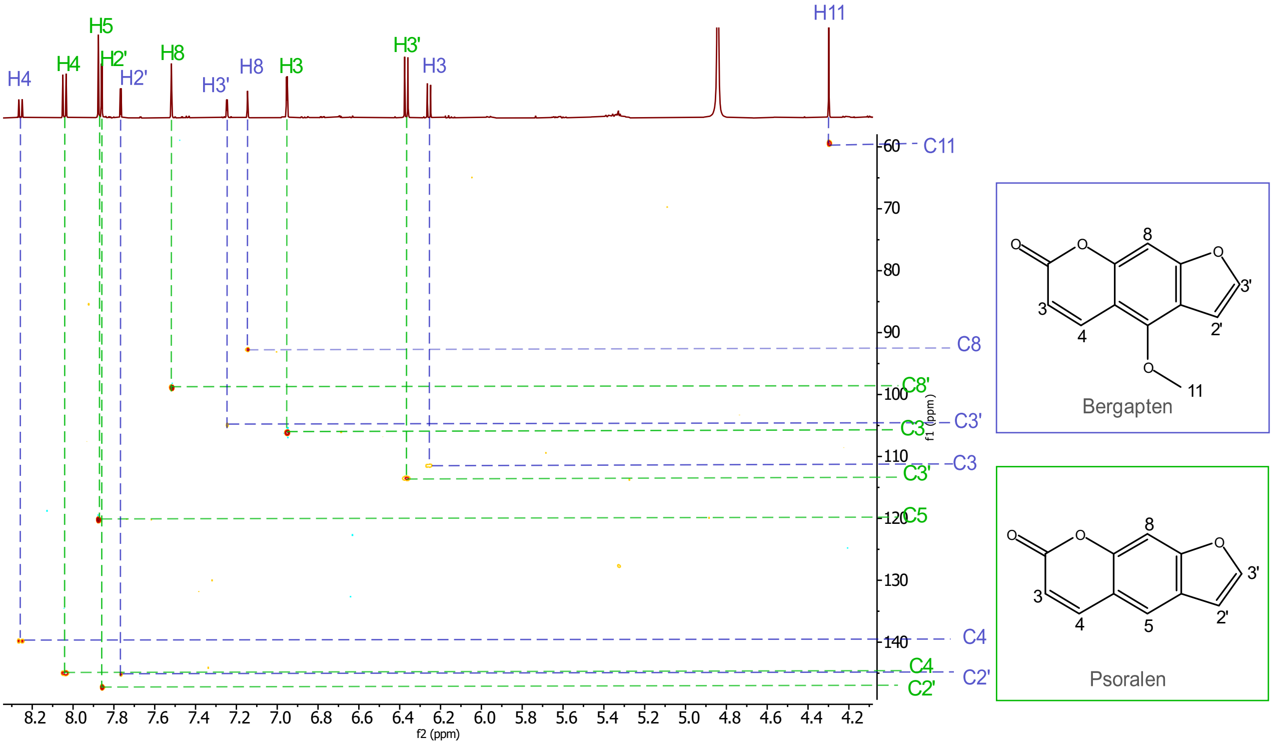

Figure S1. HSQC spectrum of *Ficus carica* chloroform fraction where ^{1}H-^{13}C correlations of bergapten (blue) and psoralen (green) are highlighted.

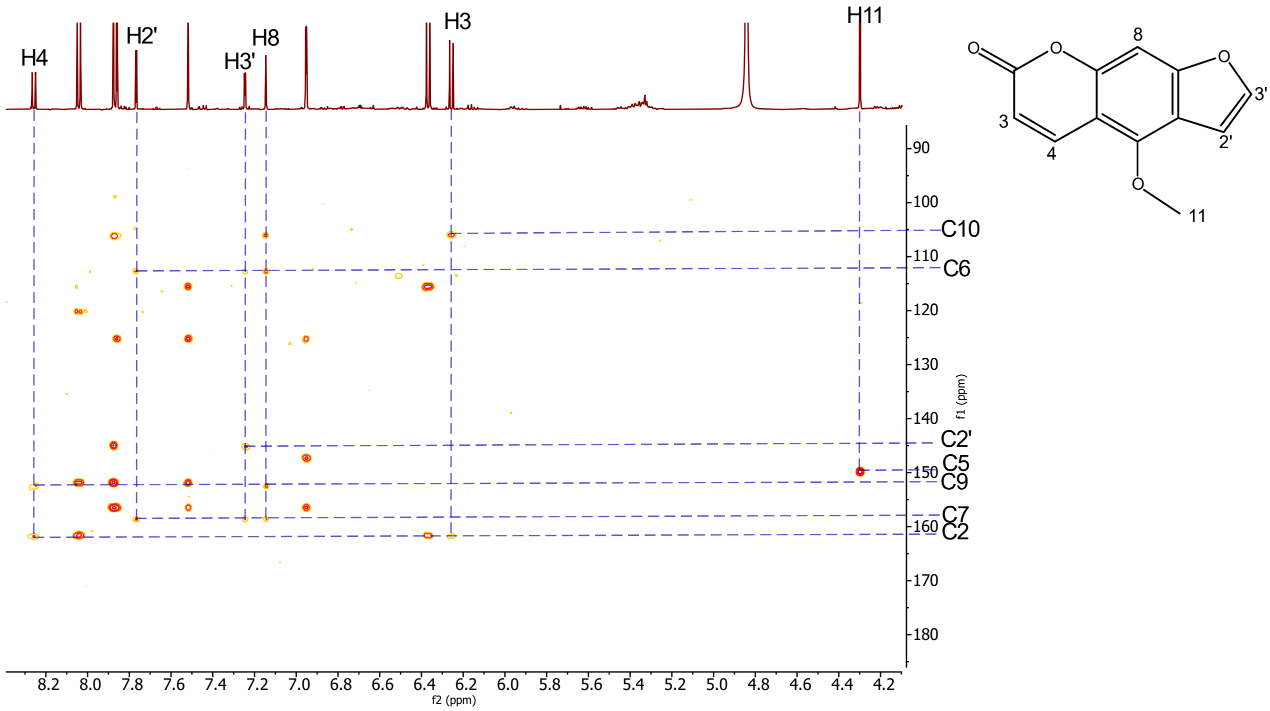

Figure S2. HMBC spectrum of *Ficus carica* chloroform fraction where ^{1}H-^{13}C correlations of bergapten are highlighted.

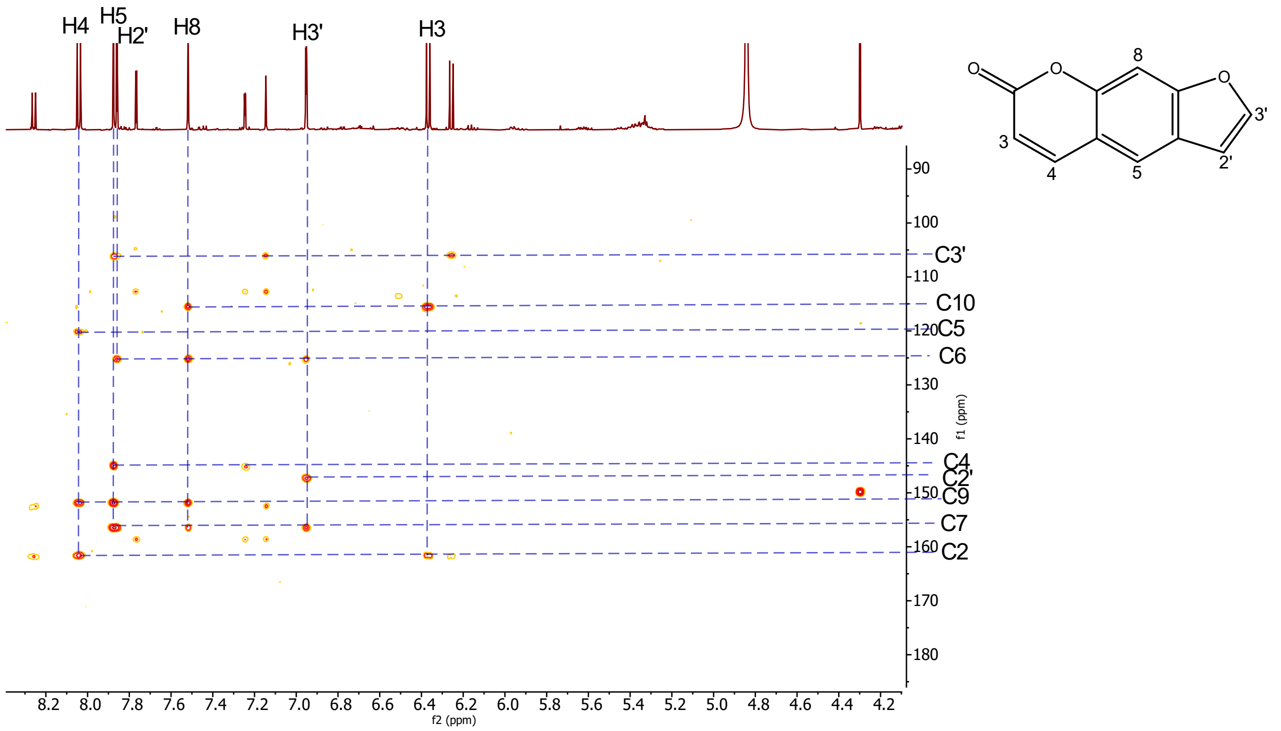

Figure S3. HMBC spectrum of *Ficus carica* chloroform fraction where ^{1}H-^{13}C correlations of psoralen are highlighted.

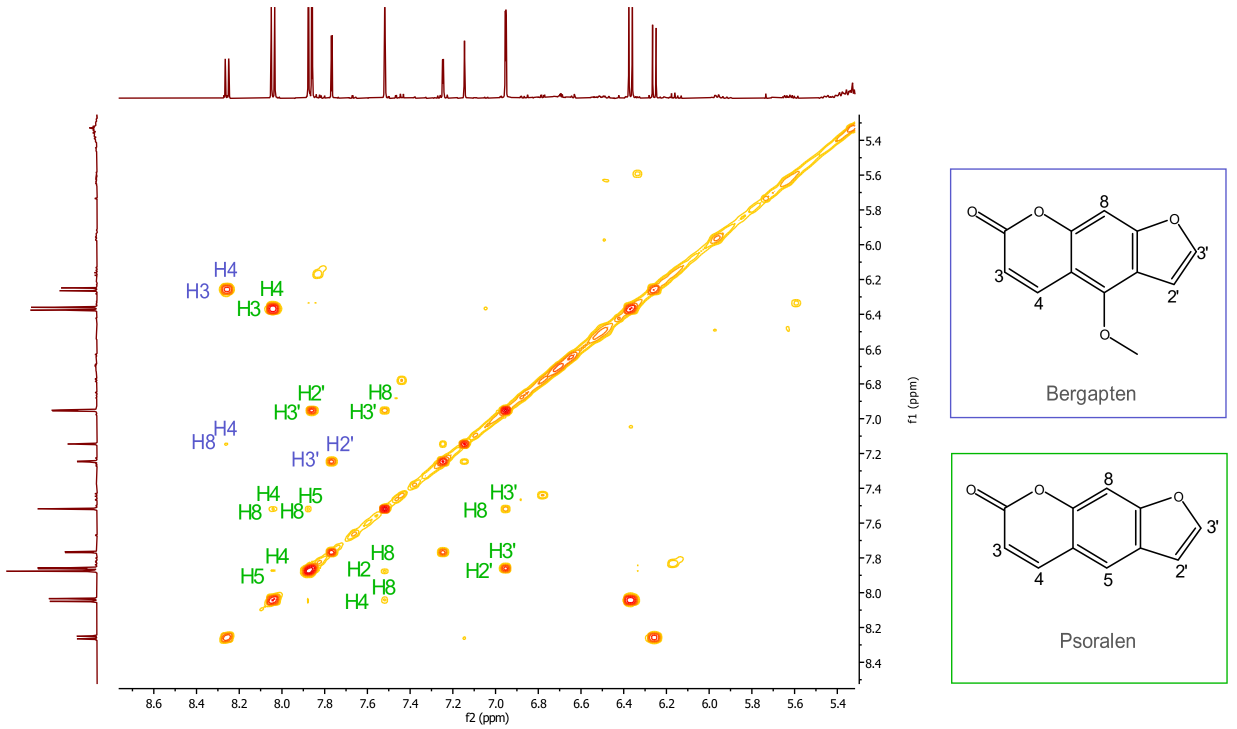

Figure S4. COSY spectrum of *Ficus carica* chloroform fraction where ^{1}H-^{1}H correlations of bergapten (blue) and psoralen (green) are highlighted.
